# The Interplay Between Personality and Clinical Syndromes in Eating Disorders: Implications for Personalized Treatment

**DOI:** 10.1002/cpp.70061

**Published:** 2025-03-17

**Authors:** Paolo Meneguzzo, Francesca Buscaglia, Anna Pillan, Enrica Bucci, Alice Garolla, Anna Marzotto, Chiara Cazzola, Roberta Castegnaro, Elisa Bonello, Patrizia Todisco

**Affiliations:** ^1^ Department of Neuroscience University of Padova Padova Italy; ^2^ Padova Neuroscience Center University of Padova Padova Italy; ^3^ Eating Disorder Unit Casa di Cura “Villa Margherita” – KOS Group, Arcugnano Vicenza Italy

**Keywords:** clinical impairment, comorbid traits, eating disorders, outcome, personality traits, treatment response

## Abstract

**Introduction:**

Eating disorders (EDs) are complex and multifactorial conditions with significant impacts on both physical and mental health. Despite advances in treatment, relapse rates remain high, highlighting the need for improved predictive models for treatment outcomes. This study aims to examine the role of personality traits and clinical syndromes, as assessed by the Millon Clinical Multiaxial Inventory‐III (MCMI‐III), in predicting treatment outcomes for individuals with EDs.

**Methods:**

A retrospective evaluation was conducted on 149 women diagnosed with EDs, receiving inpatient treatment at the Eating Disorder Unit of Casa di Cura Villa Margherita‐KOS (Arcugnano‐Vicenza) between 2020 and 2024. Participants completed the MCMI‐III at treatment initiation, with the Eating Disorder Examination Questionnaire (EDE‐Q) and Clinical Impairment Assessment (CIA) administered at baseline and discharge.

**Results:**

Regression analyses revealed that maladaptive personality traits (avoidant, dependent and histrionic) and clinical syndromes such as major depression were significant negative predictors of treatment response. In contrast, post‐traumatic stress emerged as a positive predictor of improvement. Specific personality and clinical patterns were associated with changes in symptomatology, including reductions in eating concerns, shape concerns and clinical impairment. However, not all dimensions were predictive of treatment outcomes.

**Conclusion:**

This study underscores the importance of personalized treatment approaches that account for both personality traits and clinical syndromes in individuals with EDs. Future research should explore how these factors interact over time and inform tailored therapeutic strategies, particularly for those with comorbid traits or disorders.

**Summary**

- Personality traits and clinical syndromes play a significant role in predicting treatment response in individuals with eating disorders.
- Avoidant, dependent and histrionic personality traits, along with major depression, are associated with poorer treatment outcomes.
- Post‐traumatic stress symptoms, despite their complexity, were associated with better treatment response, suggesting the importance of trauma‐informed care.
- Standard CBT‐based treatment may not be sufficient for all patients, highlighting the need for individualized interventions targeting personality features and mood disturbances.
- Identifying psychological predictors of treatment response can help clinicians personalize care and improve long‐term recovery outcomes.

## 1 Background

Eating disorders (EDs), including conditions such as anorexia nervosa (AN), bulimia nervosa (BN) and binge‐eating disorder (BED), pose a significant challenge to public health due to their complex origins, possible long‐lasting duration and profound impact on both physical and mental health (Barakat et al. 2023; Culbert et al. 2015; Santomauro et al. 2021; Treasure et al. 2020). Despite considerable progress in treatment modalities, high relapse rates persist, making the prediction of treatment outcomes a critical concern (Solmi et al. 2024). In this light, examining the psychological and personality characteristics of individuals with EDs might be essential for developing interventions that are more personalized and effective (Levinson et al. 2018, 2021; Treasure 2019).

Research and theoretical frameworks consistently highlight the influential role of personality traits in the onset, persistence and treatment outcomes of EDs (Farstad et al. 2016; Simpson et al. 2022). Different approaches have been proposed over the years to evaluate personality traits and their impacts, not only in the ED field (Martinussen et al. 2017). The Millon Clinical Multiaxial Inventory (MCMI) offers a robust system for assessing personality traits and clinical states, enabling a detailed exploration of the relationship between stable personality features and temporary psychological conditions (Choca and Grossman 2015). This dual perspective is particularly relevant for understanding EDs, as traits such as perfectionism, emotional instability and impulsivity often interact with clinical states, including depression, anxiety and obsessive‐compulsive tendencies, to shape the trajectory of the disorder (Meneguzzo, Bonello, et al. 2024; Meneguzzo, Garolla, et al. 2022; Todisco et al. 2020).

Millon's conceptualization of personality traits encompasses enduring patterns of cognition, emotion and behaviour that influence how individuals engage with their environments (Rossi and Derksen 2015). These traits span a continuum from adaptive to maladaptive. In the context of EDs, maladaptive traits—such as compulsiveness, avoidance, or narcissism—can intensify the severity of the disorder or hinder recovery efforts (Carr et al. 2021; Todisco et al. 2023). In contrast, adaptive traits, such as resilience and self‐awareness, may facilitate better engagement with treatment and improved outcomes (Coutinho et al. 2024).

Clinical states refer to temporary psychological or emotional conditions that can fluctuate over time. These states often reflect the immediate effects of the disorder on an individual's mental well‐being. For example, acute anxiety or depressive symptoms are frequently observed in individuals with EDs and can impede treatment adherence or effectiveness (Sander et al. 2021). Analysing the dynamic interaction between personality traits and clinical states is therefore crucial for identifying predictors of treatment success.

Treatments for EDs typically involve a multidisciplinary approach that addresses medical stabilization, nutritional restoration and psychological therapy (Pisetsky et al. 2020). Although these programs are comprehensive, responses to treatment can vary significantly between individuals, underscoring the need for predictive factors that inform personalized care strategies. Incorporating Millon's framework into inpatient treatment assessments may provide valuable insights into the factors driving treatment responses, ultimately guiding more effective interventions. In this perspective, this work aligns with the increasing focus on dimensional approaches in psychiatry, which emphasize understanding the continuous and interactive nature of psychological factors rather than adhering strictly to categorical diagnoses (Wildes and Marcus 2013; Williamson et al. 2005). By integrating Millon's theoretical insights into the field of EDs, this study aims to bridge the gap between personality research and clinical practice, advancing the quality of care for individuals facing these challenging conditions.

Therefore, this study investigates the predictive role of personality traits and clinical states, as defined by Millon's classification system, in treatment outcomes for individuals with EDs receiving specialized inpatient care. By examining these relationships, the study aims to contribute to the development of more individualized and effective treatment approaches. Specifically, it will explore how maladaptive personality traits and variable clinical states influence outcomes such as specific symptom reduction. Additionally, the research seeks to identify profiles of patients who may derive the greatest benefit from tailored therapeutic strategies.

## 2 Methods

For this study, a retrospective evaluation of clinical records was conducted for individuals who underwent inpatient treatment at the Eating Disorder Unit of Casa di Cura Villa Margherita‐KOS (Arcugnano‐Vicenza) between 2020 and 2024. A total of 149 women diagnosed with EDs were identified. Diagnoses were established by experienced clinicians specializing in EDs through semi‐structured clinical interviews based on DSM‐5 criteria, following standard clinical practices (Shankman et al. 2018). Among the participants, the distribution of diagnoses included AN, BN, BED and other specialized eating and feeding disorder (OSFED) reflecting the diverse presentations of EDs in this clinical population. Exclusion criteria included severe acute psychiatric comorbidities, such as psychosis or mania, that could interfere with inpatient treatment.

Written informed consent was obtained from all participants before their data was included in the study. The study protocol was approved by the Vicenza Ethics Committee (VI 47/21) as part of a longitudinal study specifically focused on treatment responses.

### 2.1 Psychological Evaluation

The psychological evaluation was performed for all participants two times with the EDEQ and the Clinical Impairment Assessment (CIA), in the first and the last week of the inpatient treatment, while the participant performed the MCMI only once, at the beginning of the treatment.

The EDE‐Q is a widely used self‐report measure designed to assess the core psychopathology associated with EDs (Luce and Crowther 1999). It evaluates key domains, including restraint, eating concern, shape concern and weight concerns. It provides both subscale scores and a global score that reflect the severity of disordered eating behaviours and attitudes. The EDE‐Q is particularly valuable in both clinical and research settings for its ability to capture subjective experiences and detect treatment‐related changes over time. In this sample, reliability was very good, with Cronbach's *α* > 0.80 across all subscales.

The CIA is a widely used self‐report questionnaire designed to measure the psychosocial impairment associated with ED symptoms (Bohn et al. 2008). It evaluates how eating habits, attitudes toward weight and shape and related behaviours impact key areas of daily functioning, such as work, social life and personal relationships. The CIA consists of 16 items rated on a four‐point scale, reflecting the severity of impairment over the past 28 days. It provides a valuable tool for clinicians and researchers to assess the broader impact of EDs beyond physical health, aiding in treatment planning and outcome monitoring. In this sample, Cronbach's *α* was 0.86.

The MCMI‐III is a psychological assessment tool designed to evaluate personality disorders and clinical syndromes, based on Theodore Millon's theory of personality (Millon and Davis 1997). It is intended for adults with existing clinical or medical concerns and is widely used in mental health, forensic and medical settings to support diagnosis, treatment planning and understanding personality dynamics. The inventory consists of 175 true/false items. It includes 24 scales, divided into personality pattern scales and clinical syndrome scales. The theoretical foundation of the MCMI‐III integrates biological predispositions, social learning and intrapsychic factors. Scores of 75 suggest clinically significant traits, while scores of 85 or higher indicate a probable disorder. In this sample, reliability was good, with Cronbach's *α* > 0.70 across all subscales.

### 2.2 Treatment

Treatment was based on a multidisciplinary cognitive‐behavioural approach tailored for individuals with EDs (Halmi 2009; Linardon and Brennan 2017; Todisco et al. 2020). The program combined psychological, nutritional and medical interventions to address both behavioural and emotional aspects of the disorder. Individual cognitive‐behavioural therapy (CBT) sessions were conducted weekly, focusing on cognitive restructuring, emotion regulation and relapse prevention. Patients also participated in daily group therapy sessions, which included psychoeducation and skills‐based training aimed at improving distress tolerance, interpersonal functioning and self‐awareness.

Nutritional rehabilitation was an integral part of the program, with structured meal planning and supervised eating guided by registered dietitians. Exposure therapy for fear foods was incorporated to help patients gradually reintroduce previously avoided foods. Continuous nursing care ensured adherence to meal plans and monitored patients' medical stability throughout their stay. Family involvement was encouraged through psychoeducational interventions designed to improve support dynamics and communication.

In addition to these core elements, the treatment included targeted adjunctive interventions based on individual needs. Mindfulness‐based strategies were introduced to improve emotion regulation, particularly in patients with high anxiety or obsessive thoughts around food. Sensorimotor therapy was available for individuals with a history of trauma, helping them reconnect with their bodily sensations in a safe and regulated way. Cognitive remediation therapy (CRT) was offered to those with cognitive rigidity, a common feature in EDs, while emotion skills training (CREST) was provided to enhance emotional awareness and processing. When necessary, pharmacological treatment was included as part of the psychiatric care plan, tailored to each patient's symptom profile.

This comprehensive approach ensured that both the behavioural symptoms and underlying psychological vulnerabilities were addressed, maximizing the chances of a meaningful and sustained recovery (Halmi 2009; Linardon and Brennan 2017; Meneguzzo, Bonello, et al. 2024; Todisco et al. 2020).(Meneguzzo, Bonello, et al. 2024).

Treatment response at discharge were assessed using an EDE‐Q global score of less than 2.3 points. This threshold corresponds to the mean plus one standard deviation of the normative score for the Italian population and aligns with recommendations from prior research (Meneguzzo, Antoniades, et al. 2024). Studies evaluating inpatient and outpatient CBT‐based treatments for EDs have reported response rates ranging from 30% to 50%, with variation depending on sample characteristics, treatment length and specific response criteria (Linardon and Brennan 2017; Vall and Wade 2015).

### 2.3 Statistical Analysis

The data were analysed using a combination of descriptive and inferential statistics to examine the relationships between personality traits, clinical syndromes and treatment outcomes in individuals with EDs. Descriptive statistics, including means, standard deviations and frequency distributions, were used to summarize the demographic and clinical characteristics of the participants. The EDE‐Q and CIA scores were assessed for changes from baseline (T0) to the end of treatment (T1) to evaluate treatment response.

To explore predictors of treatment outcomes, logistic regression analyses were conducted with treatment response (categorized as responders vs. nonresponders based on a predefined cutoff in the EDE‐Q global score) as the dependent variable. Predictor variables included the MCMI‐III personality traits and clinical syndromes. The model's goodness of fit was assessed using the chi‐square statistic (*χ* ^2^) and the Nagelkerke *R* ^2^ for variance explanation.

Additionally, linear regression analyses were performed to identify predictors of changes (Δ) in EDE‐Q subscales (such as restraint, eating concerns, shape concerns and weight concerns) and CIA scores. The Δ values were evaluated by subtracting T0 scores from T1 scores. The level of statistical significance was set at *p* < 0.05 for all analyses. Data analysis was conducted using SPSS 25.0 (IBM).

## 3 Results

### 3.1 Clinical and Demographic Description

The study sample consisted of 149 cisgender women, predominantly diagnosed with AN, which is consistent with the inpatient recruitment context. Table 1 provides an overview of the participants' demographic and clinical characteristics.

**TABLE 1:** Demographic characteristics of the participants.

Age, years	25.96 (8.67)	18.00–55.00
BMI, kg/m^2^	21.06 (9.26)	11.45–54.77
Diagnosis		
Anorexia nervosa	82	55%
Bulimia nervosa	35	23%
Binge eating disorder	18	12%
OSFED	14	10%

*Note:* The table presents the means, standard deviations, and ranges for continuous data, as well as frequencies for categorical variables. *Abbreviation*: OSFED, other specified eating and feeding disorder.

**FIGURE 1:**
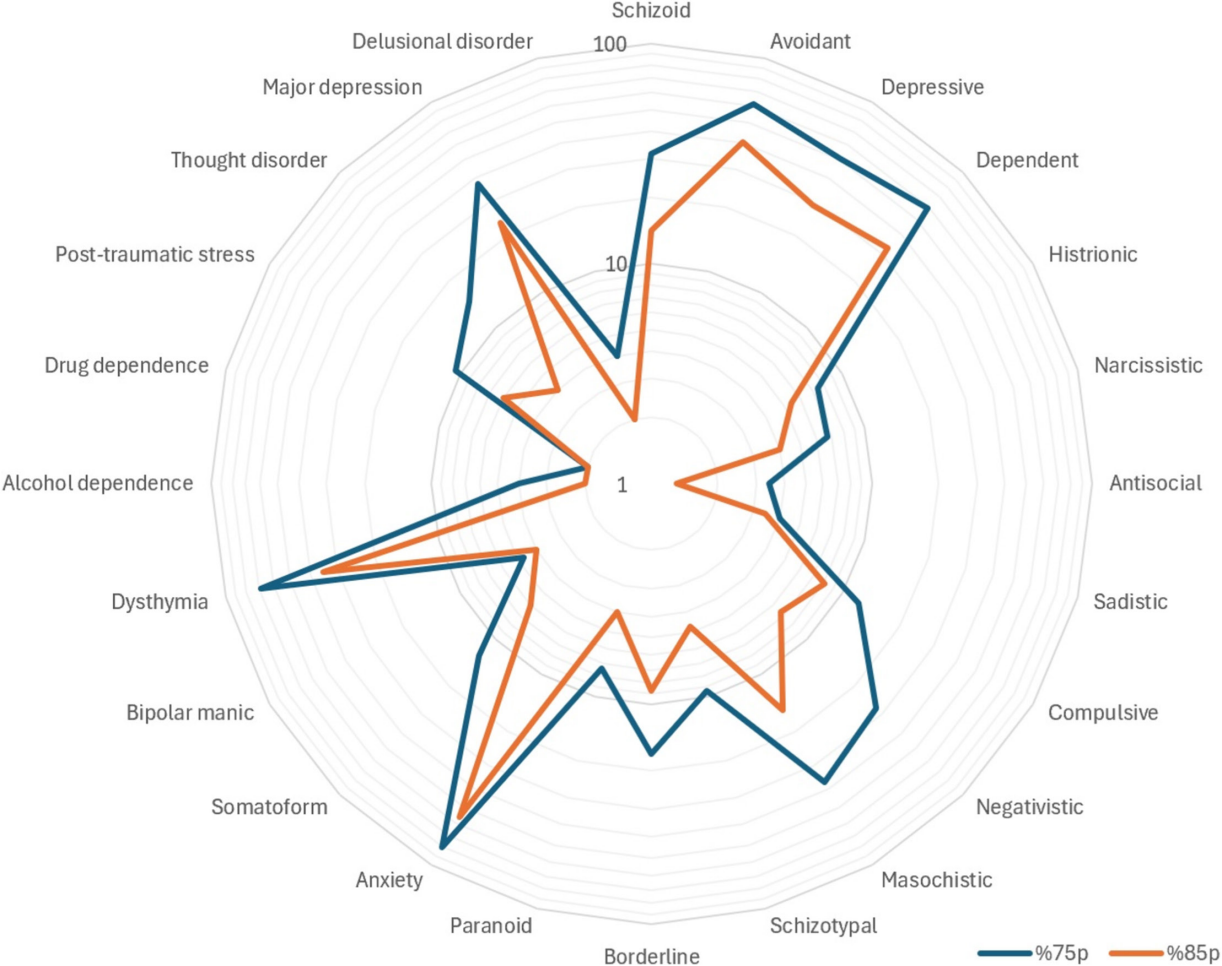
This figure displays the proportion of participants scoring above the thresholds of 75 (clinical relevance) and 85 (clinically significant) on the Millon subscales, plotted on a log10 scale to better represent the wide range of percentages. The use of the log10 scale highlights differences in subscale scores, particularly in subscales where fewer participants exceed the thresholds.

Table 2 presents the results of the MCMI‐III, organized into four main categories: Clinical Personality Patterns (58.65 ± 6.73), Severe Personality Pathology (56.50 ± 15.53), Clinical Syndromes (58.76 ± 15.34) and Severe Clinical Syndromes (54.81 ± 18.64). For each category, the table reports the mean scores and standard deviations, alongside the number and percentage of participants scoring above the clinically significant thresholds of 75 and 85 points. Within the Clinical Personality Patterns category, the highest mean score was observed for the Avoidant scale (75.67 ± 21.67), followed by the Dependent scale (73.54 ± 21.64), indicating prominent characteristics of avoidance and dependence in the sample. Conversely, the Histrionic scale displayed the lowest mean score (31.11 ± 27.18). For the Severe Personality Pathology category, the mean scores were generally lower, with the highest score observed for the Schizotypal scale (60.62 ± 15.12). Similarly, among the Clinical Syndromes, the Anxiety scale showed the highest mean (82.32 ± 20.24), followed by Dysthymia (78.09 ± 20.77). Within the Severe Clinical Syndromes category, the Major Depression subscale had the highest mean score (65.89 ± 26.11). See Figure 1 for a graphical representation of the subscale scores.

**TABLE 2:** The Millon Clinical Multiaxial Inventory‐III results.

Category	Diagnostic scales	Means	Stand. deviation	*n* > 75 points	*n* > 85 points
Clinical personality patterns	Schizoid	65.51	19.09	47 (31.5%)	21 (14,1%)
	Avoidant	75.67	21.67	91 (61.1%)	60 (40,3%)
	Depressive	71.71	21.37	76 (51.0%)	43 (28,9%)
	Dependent	73.54	21.64	88 (59.1%)	49 (32,9%)
	Histrionic	31.11	27.18	11 (7.4%)	8 (5,4%)
	Narcissistic	39.21	22.70	10 (6.7%)	6 (4,0%)
	Antisocial	49.13	17.93	5 (3.4%)	2 (1,3%)
	Sadistic	50.54	20.77	6 (4.0%)	5 (3,4%)
	Compulsive	57.41	20.43	18 (12.1%)	12 (8,1%)
	Negativistic	64.07	18.34	41 (27.5%)	10 (6,7%)
	Masochistic	67.21	17.73	55 (36.9%)	23 (15,4%)
Severe personality pathology	Schizotypal	60.62	15.12	14 (9.4%)	7 (4,7%)
	Borderline	49.10	26.61	25 (16.8%)	13 (8,7%)
	Paranoid	59.77	19.55	11 (7.4%)	6 (4,0%)
Clinical syndromes	Anxiety	82.32	20.24	120 (80.5%)	83 (55,7%)
	Somatoform	51.54	23.18	19 (12.8%)	9 (6,0%)
	Bipolar manic	50.30	22.74	7 (4.7%)	6 (4,0%)
	Dysthymia	78.09	20.77	102 (68.5%)	52 (34,9%)
	Alcohol dependence	54.60	19.40	6 (4.0%)	3 (2,0%)
	Drug dependence	48.50	21.63	3 (2.0%)	3 (2,0%)
	Post‐traumatic stress	45.96	30.46	16 (10.7%)	9 (6,0%)
Severe clinical syndromes	Thought disorder	63.77	17.92	22 (14.8%)	6 (4,0%)
	Major depression	65.89	26.11	56 (37.6%)	35 (23,5%)
	Delusional disorder	34.77	27.03	6 (4.0%)	3 (2,0%)
Total MCMI‐III		57,93	10.13		

Table 3 illustrates the changes in ED symptomatology and related impairment over time.

**TABLE 3:** The Eating Disorder Examination Questionnaire (EDE‐Q) and the Clinical Impairment Assessment (CIA) scores at the beginning of the inpatient treatment (T0) and at discharge (T1).

	T0	T1	*t* *p*
EDE‐Q			
Restraint	3.76 (1.78)	1.09 (1.14)	16.12 < 0.001
Eating Concern	3.80 (1.34)	1.88 (1.26)	12.27 < 0.001
Shape Concern	5.09 (1.19)	4.11 (1.53)	7.89 < 0.001
Weight Concern	4.60 (1.36)	3.24 (1.64)	9.04 < 0.001
Total score	4.35 (1.09)	2.60 (1.21)	14.97 < 0.001
CIA	35.55 (9.69)	21.53 (11.71)	13.09 < 0.001

*Note:* The table reports means and standard deviations.

### 3.2 Analysis of Treatment Response Predictors

The analysis of the reduction in the EDE‐Q global score showed that 48 participants (32.2%) could be considered responders, while the majority (101, 67.8%) were classified as nonresponders due to insufficient reduction in specific psychopathological concerns. No significant differences emerged in the distribution of diagnoses between responders and nonresponders (*χ* ^2^ = 2.45, *p* = 0.653).

The logistic regression analysis of treatment response indicated that the model including MCMI‐III predictors was statistically significant (*χ* ^2^ = 52.61, *p* = 0.001) and explained a relevant amount of variance (*R* ^2^ = 0.42). It identified several predictors of nonresponse to treatment. Negative predictors of treatment response included the presence of avoidant (*β* = −0.04, S.E. = 0.02), dependent (*β* = −0.03, S.E. = 0.02) and histrionic (*β* = −0.04, S.E. = 0.02) personality traits, as well as major depression (β = −0.05, S.E. = 0.02). These factors were associated with a lower likelihood of improvement. In contrast, post‐traumatic stress disorder (*β* = 0.02, S.E. = 0.01) emerged as a positive predictor, indicating a greater likelihood of treatment response. See Table 4 for details.

**TABLE 4:** Predictors of treatment response and non‐response.

Scales	*p*	*B*	95% CI
Schizoid	0.246	0.98	0.94–1.02
Avoidant	0.044	0.96	0.92–0.99
Depressive	0.436	0.99	0.96–1.02
Dependent	0.047	0.97	0.92–0.99
Histrionic	0.048	0.96	0.94–0.99
Narcissistic	0.123	1.03	0.99–1.07
Antisocial	0.109	0.95	0.89–1.01
Sadistic	0.175	1.02	0.99–1.06
Compulsive	0.527	1.01	0.98–1.05
Negativistic	0.283	1.02	0.98–1.07
Masochistic	0.165	1.03	0.99–1.08
Schizotypal	0.251	1.03	0.98–1.08
Borderline	0.977	1.00	0.97–1.03
Paranoid	0.135	0.98	0.94–1.01
Anxiety	0.168	0.97	0.93–1.01
Somatoform	0.318	1.02	0.98–1.05
Bipolar manic	0.196	0.98	0.95–1.01
Dysthymia	0.295	1.02	0.98–1.06
Alcohol dependence	0.286	1.02	0.97–1.05
Drug dependence	0.665	1.01	0.97–1.05
Post‐traumatic stress	0.048	1.02	1.01–1.05
Thought disorder	0.181	1.04	0.98–1.09
Major depression	0.015	0.95	0.92–0.99
Delusional disorder	0.230	0.99	0.96–1.06

Using the four MCMI‐III categories as predictors, we found a significant regression model (*χ* ^2^ = 14.63, *p* = 0.006, *R* ^2^ = 0.13) for treatment response, in which only the Severe Clinical Syndromes category emerged as a predictor (*B* = 0.96, *p* = 0.015 [0,93–0,99]), with negative effects on outcomes.

Then, we performed several linear regression analyses to examine the MCMI‐III subscales as potential predictors of changes in ED psychopathology and clinical impairment. We found that ΔCIA was predicted by depressive (*t* = −2.69, *p* = 0.008, B = −0.38) and major depression (*t* = 3.50, *p* = 0.001, *B* = 0.67) subscales, with a significant model fit (*F* = 1.81, *p* = 0.024, *R* ^2^ = 0.15). ΔEDE‐Q global score was predicted by dysthymia (*t* = −2.27, *p* = 0.025, *B* = −0.37), drug dependence (*t* = −2.46, *p* = 0.016, *B* = −0.43), post‐traumatic stress (*t* = −2.53, *p* = 0.013, *B* = −0.34) and major depression (*t* = 3.36, *p* = 0.001, *B* = 0.65) subscales, with a significant model fit (*F* = 1.94, *p* = 0.013, *R* ^2^ = 0.16). ΔRestraint was not predicted by any of the variables examined (*F* = 1.28, *p* = 0.201). ΔFood concerns was predicted by dysthymia (*t* = −2.64, *p* = 0.004, *B* = −0.39) and major depression (*t* = 4.04, *p* < 0.001, *B* = 0.75) subscales, with a significant model fit (*F* = 2.15, *p* = 0.005, *R* ^2^ = 0.19). Finally, ΔShape concerns (*F* = 1.47, *p* = 0.096) and ΔWeight concerns (*F* = 1.09, *p* = 0.369) were not predicted by any of the variables included.

## 4 Discussion

This study aimed to examine the role of personality traits and clinical states, as assessed through the MCMI‐III, in predicting treatment outcomes for individuals with EDs undergoing inpatient treatment. The findings suggest that personality traits, particularly maladaptive traits associated with the MCMI‐III, and specific clinical syndromes might play a significant role in determining treatment response and symptom reduction over the course of inpatient care.

The analysis of treatment response revealed that a significant proportion of the samples were classified as nonresponders, highlighting the challenge of achieving consistent treatment outcomes in EDs (Linardon et al. 2017, 2019). Previous studies suggest that while CBT remains the gold standard for EDs, its efficacy is often moderated by individual psychological factors such as personality traits, affect regulation difficulties and trauma history (Levinson et al. 2021; Meneguzzo, Marzotto, et al. 2024; Vall and Wade 2015). Indeed, current treatment approaches still yield suboptimal outcomes, with a significant proportion of individuals experiencing persistent residual symptoms or requiring repeated treatment episodes (Solmi et al. 2024). Additionally, comorbid psychiatric conditions, including depression, anxiety and post‐traumatic stress disorder, have been shown to negatively impact recovery trajectories, potentially contributing to the heterogeneity observed in treatment response (Convertino and Mendoza 2023; Monteleone 2021). This is consistent with existing literature that suggests high relapse rates and variable responses to treatment among individuals with EDs, emphasizing the need for tailored interventions (Khalsa et al. 2017; Sala et al. 2023). Indeed, current approaches for EDs still show poor outcome rates, with frequent relapses and long‐lasting durations (Solmi et al. 2024). Our results support the idea that limiting interventions to a symptom‐driven therapeutic approach may not be sufficient for achieving optimal outcomes, due to the significant role played by other specific psychological factors (Byrne and Fursland 2024). The logistic regression analysis indicated that certain personality traits, including avoidant, dependent and histrionic traits, as well as major depression, were negative predictors of treatment response. These findings align with previous research that has identified maladaptive personality traits and comorbid depression as barriers to successful treatment outcomes in ED populations (Hambleton et al. 2022; Meneguzzo et al. 2021; Vall and Wade 2015). These traits may interfere with treatment engagement, emotional regulation and interpersonal functioning, thus complicating the recovery process. They might also exacerbate negative emotional states that could interfere with interpersonal dynamics, such as the therapeutic alliance (Todisco et al. 2023).

Interestingly, post‐traumatic stress subscale emerged as a positive predictor of treatment response. This finding is somewhat counterintuitive, as post‐traumatic stress is typically associated with chronicity and greater severity in various psychiatric disorders (Cloitre et al. 2009; Meneguzzo, Mancini, et al. 2022; Monteleone 2021; Scharff et al. 2021). However, it may suggest that individuals with a history of trauma could benefit from interventions specifically designed to address trauma‐related issues. Notably, the treatment protocol employed in this study incorporated trauma‐informed care and trauma‐focused therapies, which may have contributed to this positive outcome as reported in previous studies (Brewerton 2019; Day et al. 2024). By integrating such approaches, which focus on understanding and addressing the impact of trauma, treatment may help reduce emotional dysregulation and enhance engagement with other therapeutic interventions. This highlights the value of including trauma‐informed strategies in the multidisciplinary treatment of EDs, as they may improve treatment adherence, foster better therapeutic alliance and ultimately lead to more favourable recovery outcomes for individuals with comorbid PTSD.

Interestingly, despite the positive association between post‐traumatic stress and treatment response, this variable emerged as a negative predictor of overall symptom reduction when examining changes in EDE‐Q scores. This discrepancy suggests a nuanced relationship between trauma‐related symptoms and treatment outcomes. On the one hand, trauma‐informed care may enable individuals to achieve critical treatment thresholds. On the other hand, the residual impact of trauma‐related emotional dysregulation or cognitive distortions might limit the magnitude of symptom reduction across the entire treatment course. This highlights the need for a comprehensive approach to addressing trauma, as its impact on various aspects of recovery may vary based on the measures used to assess treatment outcomes, underscoring the importance of conducting further studies to clarify these complex relationships (Convertino and Mendoza 2023; Rienecke et al. 2021).

In further examining the predictors of symptom reduction, the study found that depressive and major depression subscales were significant predictors of improvement in both the CIA and the EDE‐Q scores. This suggests that targeting mood disturbances in ED treatment could play a critical role in facilitating symptom reduction and improving psychosocial functioning. In particular, the significant association between depressive symptoms and reductions in food concerns and clinical impairment underscores the importance of addressing comorbid mood disorders in the treatment of EDs (Berkman et al. 2007).

Conversely, dysthymia, drug dependence and post‐traumatic stress emerged as significant negative predictors of symptom reduction, particularly in relation to changes in EDE‐Q scores and food concerns. These findings underscore the complex relationship between psychiatric comorbidities and the psychopathology of EDs, revealing how conditions such as depression, substance use and trauma can hinder progress in ED treatment. This highlights the necessity for comprehensive treatment approaches that go beyond addressing the core ED symptoms (Hambleton et al. 2022). Clinicians should consider the broader mental health context, incorporating strategies to manage mood disorders, substance use and trauma‐related symptoms in order to optimize treatment outcomes. The interaction between these factors suggests that effective interventions must be multidimensional, targeting both the specific ED pathology and the comorbid psychiatric conditions that can influence recovery trajectories.

No significant predictors were identified for changes in restraint, shape concerns or weight concerns. These results suggest that some dimensions of ED psychopathology may be less responsive to treatment in this sample, which could be due to the chronic nature of these concerns or the fact that they are more deeply rooted in the individual's personality structure. It is possible that these concerns require more intensive, long‐term interventions or that additional factors, such as social influences, play a larger role in shaping these dimensions.

A key finding of this study was the identification of severe clinical syndromes, particularly major depression, as a significant negative predictor of treatment outcomes. This aligns with findings from previous research suggesting that severe clinical comorbidities, such as major mood disorders, can profoundly influence the course of EDs. These results may underline the presence of an interplay between these conditions, with each potentially influencing the others in a way that complicates treatment outcomes.

The findings of this study contribute to the expanding body of literature examining the role of personality traits and clinical syndromes in the prognosis of EDs. By identifying specific personality patterns and clinical states that predict treatment outcomes, this study emphasizes the critical importance of personalized treatment approaches that consider individual psychological profiles. Tailored interventions targeting maladaptive personality traits and mood disturbances may enhance treatment engagement, facilitate symptom reduction and promote overall recovery. However, it is important to acknowledge the limitations of this study. The retrospective design limits the generalizability of the findings, and the inclusion of only cisgender women further restricts the applicability of the results to more diverse populations. Additionally, the MCMI‐III is a self‐report measure, which may be subject to response biases, including social desirability and inaccuracies in self‐reporting, and future studies should include an evaluation of the personality traits with a structured interview. Furthermore, the questionnaire has been critiqued for certain psychometric limitations, including the potential overlap between subscales, given its dimensional goals and structure.

## 5 Conclusion

In conclusion, this study underscores the significant role of personality traits and clinical syndromes in predicting treatment outcomes for individuals with EDs. Specifically, maladaptive personality traits such as avoidant, dependent, and histrionic patterns, along with major depression, were identified as negative predictors of treatment response, while post‐traumatic stress emerged as a positive predictor. The findings highlight the importance of considering both personality and mood disturbances when developing treatment plans. Future research should focus on further elucidating the complex interaction between these factors over time and the potential benefits of personalized interventions targeting both ED symptoms and underlying psychological features. Additionally, exploring the role of trauma in treatment response could inform the development of more effective, trauma‐informed therapeutic strategies. This would be especially relevant for individuals with EDs and traumatic history, offering more targeted and potentially beneficial interventions.

## Author Contributions

P.M.: conceptualization, methodology, formal analyses, data collection, data curation and original writing; F.B.: investigation, data collection, writing review and editing; A.P.: investigation, data collection, writing – review and editing; E.B.:investigation, data collection, writing review and editing; A.G.: investigation, data collection, writing – review and editing; A.M.:investigation, data collection, writing – review and editing; C.C.: investigation, data collection, writing – review and editing; R.C.: investigation, data collection, writing – review and editing; E.B.: investigation, data collection, writing – review and editing; P.T.: conceptualization, methodology, funding acquisition, supervision, project administration, resources, writing – original draft and review and editing. All authors read and approved the final version.

## Ethics Statement

The authors assert that all procedures contributing to this work comply with the ethical standards of the relevant national and institutional committees on human experimentation and with the Declaration of Helsinki of 1975, as revised in 2008. The research protocol obtained specific approval from the Vicenza Ethics Committee (47/21).

## Conflicts of Interest

The authors declare no conflicts of interest.

## Data Availability

Data supporting the findings of this study are available from the corresponding author, upon reasonable request.
